# Factors associated with self-rated health in primary care

**DOI:** 10.1080/02813432.2018.1499590

**Published:** 2018-08-23

**Authors:** Thomas Mildestvedt, Vibeke V. Herikstad, Ida Undheim, Bjørn Bjorvatn, Eivind Meland

**Affiliations:** Department of Global Public Health and Primary Care, University of Bergen, Bergen, Norway

**Keywords:** Family practice, primary health care, self-rated health, sleep problems, somatic health complaints

## Abstract

**Background:** Self-rated health (SRH) measures one’s current general health and is a widely used health indicator. Sleep problems, somatic health complaints, and unmet needs in interpersonal relationships are suspected to influence SRH, but studies in primary health care settings are sparse.

**Objective:** To examine the associations between patients’ self-rated health and their sleep problems, somatic health complaints, and unmet needs in interpersonal relationships.

**Design:** We collected data via questionnaires for this cross-sectional study from general practice.

**Setting:** Primary health care in Norway.

**Subjects:** 1302 consecutive patients participated.

**Main outcome measures:** The questionnaire included a single question about SRH, the Bergen Insomnia Scale (BIS), five questions on somatic health complaints, and three questions from the Basic Psychological Needs Scale (BPNS) pertaining to the relationships domain. We analyzed our data using ordinal logistic regression models.

**Results:** Our response rate was 74%. The prevalence of fair/poor SRH was 26%, with no gender differences. We revealed a significant association between increasing age and reduced SRH. The study showed that sleep problems and somatic health complaints were strongly associated with SRH, and unmet needs in relationships were also significantly and independently associated with reduced SRH in a full model analysis.

**Conclusion:** Sleep problems, somatic health complaints, and unmet needs in interpersonal relationships were all associated with reduced SRH. These factors are all modifiable and could be managed both within and outside a primary care setting in order to improve SRH.

**Key Points**
- There was a high prevalence of reduced SRH in clinical general practice
- Sleep problems, somatic health complaints, and unmet needs in interpersonal relationships were all associated with reduced SRH
- These predictors are all modifiable with a potential to improve SRH

## Introduction

Self-rated health (SRH) measures a person’s current general health and is a widely-used health indicator, typically assessed by a single question. It is shown to predict mortality even after adjusting for variables such as age, socio-economic status, as well as other medical, psychological, and behavioral elements [1]. A broad composite of factors is included in SRH reflecting medical, social, and personal attributes [2]. Somatic symptoms, such as musculoskeletal pain, stomach pain, or headache are associated with lower SRH during adolescence and early adulthood [2]. A cross-sectional study showed a high prevalence of chronic pain that increased with age, and those reporting chronic pain had a much higher risk of reporting reduced SRH [3].

Sleep problems are more common among those experiencing high financial strain, high psychological distress, and negative social interactions [4]. Sleep problems, including both short and long sleep duration, are associated with reduced SRH [5].

People who experience social isolation more often report low SRH compared to those with strong social ties [6]. The need for relatedness is one of the components of the Basic Psychological Needs Scale (BPNS), which also encompasses the need for competence and for autonomy [7]. The satisfaction of basic psychological needs (BPN) is related to indicators of wellness such as higher self-esteem and life-satisfaction [7]. Higher levels of socio-economic status predict higher levels of satisfaction of BPN [8]. A recent study has shown that the fulfillment of BPN serves as an explanatory variable linking socio-economic status to self-rated health [8].

In a large cross- sectional study from 14 countries outside Scandinavia pain sufferers reported unfavorable SRH compared to those without pain. This WHO study found large variations in the reports of pain and SRH suggesting more research in different settings [9]. (We are presenting data from patients in the General Practitioner´s waiting room, a population with more health complaints compared to the general population. The shift of morbidity from well-defined somatic and objectively verifiable disease to subjective ailments represents a challenge for every clinical field, and for general practice in particular. A recent study among general practitioners (GPs) in the Nordic countries revealed great variability in diagnostic assessment when faced with patients with subjective health complaints. The same GPs almost uniformly offered the same passive treatment, such as sick leave certificates [10,11]. Research points to an imbalance between chronic distress and personal coping skills which can lead to health complaints and reduced SRH [12]. Recent advances in psychotherapeutic methods emphasize that well-intended sympathy with patients´ difficulties and suffering may lead to dysfunctional coping by promoting avoidant behavior [13]. Research that can reveal associations and possible causal links between modifiable factors and SRH is therefore justified.

There are about 4,800 GPs in Norway and since 2001 all citizens have a legal right to be included on a GP´s patient list. This system facilitates continuity with patients and coordination of treatment, as GPs function as gatekeepers for referrals to specialists or hospitals, ensuring a cost-effective health care system.

With this background, we set out to examine the associations between patients’ assessment of SRH and their sleep problems, somatic health complaints, and unmet relationship needs in a primary health care setting in Norway.

## Methods

The study was based on patients’ self-reported data collected in September and October 2014. Our data came from questionnaires distributed by 6^th^ year medical students from the University of Bergen during their four-week deployment in general practice. The students or the GPs’ secretaries invited all patients who were attending the GP´s office on one or two random days to participate. Each student aimed at collecting 20 completed questionnaires during the selected day(s). Participating patients gave verbal informed consent.

The one-page questionnaire consisted of questions concerning SRH, sleep, somatic health complaints, and interpersonal relationship needs. We did not ask for any personal or identifiable information, except for patients’ gender and age. The questionnaire was distributed to patients in the GP’s waiting area before their consultation. We excluded all patients who did not report their age and those younger than 16 years because research on children need parental accept.

## Study measures

### Self-rated health

The question pertaining to self-rated general health was: “How, in general, would you rate your health – poor, fair, good, very good, or excellent?”. This question has been used to measure health for decades, with established validity [1,14]. We rescaled the question into three categories: those reporting their health as excellent and very good in the first group, those reporting their health as good in the second group, and those reporting their health as fair or poor in the third group.

### Sleep

Insomnia symptoms were reported using the Bergen Insomnia Scale (BIS) [15]. There are six items in the BIS, each with eight possible responses. The sum score provides a scale, value 0-42, and a higher score indicates a greater degree of insomnia. Good internal consistency is demonstrated by the BIS, with Cronbach’s α = 0.84 (Table 1). We applied the BIS to examine the prevalence of sleep problems in the same primary health care population in a previous study [16].

**Table 1.** The independent variables (self-rated health, sleep and satisfaction with interpersonal relationships) used in the analyses, including response options, missing values, valid response, Cronbach’s alpha, mean values and min/max values for composite scores.

Variables	Response options	Valid response	Cronbach’s alpha	Mean (SD)	Range
Self-rated health					
How, in general, would you rate your health?	5 (1–5)	1291		2.89 (1.02)	1–5
Sleep					
Bergen Insomnia scale (sum score of six items^a^)	43 (0–42)	1282	0.84	12.51 (11.01)	0–42
Somatic health complaintsin the past 6 months, how often have you experienced:			0.75	2.02 (0.96)	1–5
Headache	5 (1–5)	1278			
Stomach ache	5 (1–5)	1252			
Backache	5 (1–5)	1260			
Neck and shoulder pain	5 (1–5)	1276			
Hip pain	5 (1–5)	1276			
Satisfaction with interpersonal relationships			0.94	4.32 (0.87)	1–5
Those who I am usually with I consider to be my friends	5 (1–5)	1292			
Those who I am with care about me	5 (1–5)	1287			
Those who I am with are quite nice to me	5 (1–5)	1289			

a Sum-score is valid (prevalence of single-item-missing for each item between 2.2% and 3.6%).

### Somatic health complaints

We measured somatic health complaints using a five-item scale, with a Cronbach´s α = 0.75. The questions on health complaints are a slight modification of the Health Behavior in School-aged Children (HBSC) symptom checklist [17], used in a WHO collaborative cross-national survey. The questions are described in Table 1, and the patients were asked to answer on a 5 point Likert scale from “rarely or never” to “about every day”.

### Relatedness (satisfaction with interpersonal relations)

We used three questions from the Basic Psychological Needs Scale (BPNS) to measure satisfaction with relatedness [18]. In this 21 item questionnaire 9 items encompassed relatedness. Diseth et al further developed this instrument to a six item questionnaire [19]. We selected the three questions with the highest factor loadings among the six items (analysis not shown). This scale proved valid and reliable in earlier studies in a Norwegian setting [19]. Table 1 presents the three questions from the scale. The internal consistency was satisfactory with a Cronbach’s α = 0.94.

### Statistics

We stratified our data into four age groups: 16-30, 31-45, 46-62, and 63-100 years, and used these groupings for cross-tabulation in Table 2. Age in years was entered in all regression models as a continuous variable. Sleep problems, somatic health complaints, and unmet needs in interpersonal relationships were analysed by sum- or mean-scores as indicated in Table 1. We tested reliability by calculating the Cronbach’s α for each construct.

**Table 2.** Cross-tabulation of self-rated health (SRH) in different age groups and gender, including the total number of responses in each SRH category.

	SRH			
	Excellent/very good *n* (%)	Good *n* (%)	Fair/poor *n* (%)	Total *n* (%)
Total	453 (35)*	503 (39)	335 (26) #	1291 (100)
Age groups				
16–30 years	137 (47)	98 (34)	55 (19)	290 (100)
31–45 years	124 (39)	120 (38)	72 (23)	316 (100)
46–62 years	99 (30)	130 (39)	104 (31)	333 (100)
63–100 years	89 (26)	154 (44)	104 (30)	347 (100)
Gender				
Male	154 (33)	191 (41)	119 (26)	464 (100)
Female	295 (36)	311 (38)	216 (26)	822 (100)

*n* = number of respondents.

* 108 (8) “excellent” # 80 (6) “poor.

Sleep problems, somatic health complaints, and unmet needs in interpersonal relationships were rescaled and standardized to compare the different independent variables. This also allowed us to compare our results with recent literature using similar standardized statistics. Each scale was based on three to six items, with response categories from one to five, or zero to seven. We recoded unmet needs in interpersonal relationships to safeguard that the variables in all three categories yielded the same direction: increasing values indicated increased impairment. The scale mean score was rescaled as (mean-1)/(k-1), where k is the number of possible response options, giving a rescaled score from 0 (best) to 1 (worst). Respondents who answered 50% or more of the questions in each construct were included. Finally, each scale score was normalized by dividing it by its standard deviation.

Analysing SRH, we applied cut-offs between excellent/very good; good; and fair/poor. We used the concept of impairment when reporting the odds for each of these cut-offs. Ordinal logistic regression analyses were performed with the three-level SRH as a dependent variable. We performed the ordinal logistic regression analyses unadjusted with one independent variable at a time as shown in Table 3, and then adjusted for age and gender. Finally, we performed a multiple ordinal regression analysis, with all the independent variables in the model simultaneously, as shown in Table 4.

**Table 3.** Unadjusted and adjusted ordinal logistic regression analyses of the relationship between self-rated health (SRH*) and the independent variables, adjusting for age and gender.

Variables	Unadjusted			Adjusted		
	OR	*p*-value	95% CI	OR	*p*-value	95% CI
Female	0.95	0.62	0.77; 1.17			
Age^a^	1.02	<0.01	1.01; 1.02			
Sleep problems^a^	2.25	<0.01	2.01; 2.52	2.42	<0.01	2.15; 2.72
Somatic health complaints^c^	2.71	<0.01	2.40; 3.07	2.90	<0.01	2.56; 3.30
Unmet needs for interpersonal relationships^c^	1.26	<0.01	1.13; 1.39	1.21	<0.01	1.08; 1.35

* SRH is divided in three levels, higher OR indicates reduced SRH.

Entered in the analyses as: ^a^Years, ^b^Sum-score of BIS, ^c^Mean-score.

**Table 4.** Full model ordinal logistic regression analysis for the relationship between self-rated health (SRH*) and the independent variables.

Variables	Adjusted			Explained variance (Nagelkerke)
	OR(a)	*p*-value	95% CI	
Gender	0.73	0.01	0.58; 0.93	
Age	1.02	<0.01	1.02; 1.03	
Sleep problems	1.81	<0.01	1.60; 2.06	
Somatic health complaints	2.33	<0.01	2.03; 2.67	
Unmet needs for interpersonal relationships	1.16	0.01	1.04; 1.30	
Explained variance				0.34

* SRH is divided in three levels, higher OR indicates reduced SRH.

Two-sided *p*-values <0.05 were considered significant. Results from the analyses were reported as an odds ratio (OR) for the effect on reduced SRH, *p*-values, and 95% confidence intervals for OR. We used SPSS version 22.0 in the analysis.

## Results

During deployment in general practice, 66 of 69 medical students collected data for the study. We calculated a response rate of 74%, based on reports from 45 students. The other 21 students did not report response rates. This was mainly due to a lack of control over how many patients in their practice were invited to participate. After exclusion, the total number of participants eligible for analysis was 1302. On average, each student collected data from 20 patients (range 4-33). The mean age of participants was 48.3 (SD 18.6) years (range 16-94). More women (64.1%) than men participated in the study.

Table 1 shows descriptive data and reliability of the outcome and the independent variables. As shown in Table 2, a total of 26% of the respondents reported their SRH health as fair/poor, with no gender differences. The proportion of people who reported fair/poor SRH increased with increasing age.

The unadjusted ordinal logistic regression analysis presented in Table 3 shows a significant association between age and reduced SRH, with the odds for reduced SRH increasing by 0.02 for every aging year. We revealed significant associations between sleep problems, somatic health complaints, and unmet needs in interpersonal relationships, and reduced SRH (Table 3).

Table 4 presents the association between the different variables and reduced SRH in a full ordinal logistic regression model with all variables entered in the analysis. In the full model, adjusting for the interrelation between the independent variables, the associations with reduced SRH were slightly attenuated, but still significant, for all predictor variables. In the final model, age and female gender were also significantly associated with reduced SRH.

The explained variance was high (0.34), manifesting the validity of the model and the relevance of the chosen predictors.

## Discussion

Our study revealed that the clinical population in general practice is characterized by a high prevalence of reduced SRH. We also identified modifiable factors such as sleep problems, somatic health complaints, and unmet needs in interpersonal relationships, that had strong and statistically significant associations with patients’ SRH. These associations persisted for all variables in a full model analysis. We do not know, however, the direction of the associations; they are probably bidirectional. SRH decreased with increasing age and female gender according to the full model analysis.

Important strengths of our study are the large sample of patients from general practice and a satisfactory response rate, which safeguarded the external validity of our findings.The questionnaire included information on a broad spectrum of factors suspected to be associated with SRH. It consisted of well-established scales and questions that have proved to be reliable and valid in earlier studies. The fact that most respondents answered all questions was an indication that the questionnaire was easy for patients to interpret.

We lacked information about the response rates from 21 practices because the number of invited patients was not recorded, and this is a limitation. In addition, data from questionnaires are vulnerable to both recall and methodological bias [20]. It is also important to bear in mind that answers to questionnaires are easily influenced by negative affectivity. Self-reporting of illness is open to systematic misreporting of personal health information and for increasing the importance of the personal psychological traits of individuals when it comes to assessing levels of poor sleep, unmet relationship needs, and somatic health complaints.

However, using the BPN to assess satisfaction has proved to be relevant for subjective health complaints [8]. In our study the construct comprised of three items, each formulated in a way that predisposes for a floor effect. We revealed a great skewedness toward the two lowest categories, these accounting for 90% of the responses. This makes statistical interpretation difficult.

The population in general practice is characterized by a high prevalence of subjective ill-being. We lack information on the prevalence of SRH in this population, and maintain therefore, that this finding is novel and important for how GPs perform clinical work. Former studies revealed that, despite diagnostic confusion, GPs in western societies are uniformly liable to sick-list patients, i.e. to issue a sick leave certificate [10,11]. For individuals with subjective ill-health, our common procedures for testing, sick-listing, and appointing return visits may be counter-therapeutic and provide little reassurance. On the contrary, they may amplify symptoms and sick role identification, with increased worry and anxiety as a result [13]. Engaging with health services and establishing medical diagnoses are associated with lower SRH [21].

Our results are in line with findings in a Finnish study linking chronic pain to reduced SRH [3]. We found a high prevalence of fair/poor SRH in our study sample (19–31% dependent on age) compared with other Scandinavian study populations of similar age and gender profiles (7.6% and 3.3-8.7% dependent on age and gender) [3,22]. A study in a similar population found that insufficient sleep was associated with poor SRH [23]. In addition, other European studies reported associations between poor social ties and lower SRH [6]. These studies are all population based and it is likely that patients in a GP`s waiting room would report more health complaints and accordingly lower SRH.

A cross-sectional study can only show associations between the independent and dependent variables and we cannot confirm the direction of any influence. Breidablik et al. found that the independent variables with high OR in a cross-sectional study also demonstrated significant predictive ability in a longitudinal study, suggesting a causal association [2,23]. The strong associations revealed in our study for somatic health complaints and sleep problems make it likely that the predictive ability applies also for our variables. This is an important note, as sleep problems, somatic health complaints, and unmet needs in interpersonal relationships are all susceptible to improvement. Intervention studies have shown that insomnia and somatic health complaints can be improved by use of cognitive behavioral therapy, acceptance and commitment therapy, and mindfulness-based therapy [24,25]. Earlier research showed that positive changes in SRH, without changes in objective health, lowered the mortality rate over the following 30 months [14].

The high prevalence of reduced SRH in clinical general practice represents a challenge for family physicians. Our study revealed that sleep problems, somatic health complaints, and unmet needs in interpersonal relationships were all associated with reduced SRH. All of these could be modified toward improvement using a range of therapies both within and outside general practice. Therefore, clinical and preventive methods for management of these factors seem important to improve subjective health.

## Ethical approval

The study was presented to the Ethics Board (The Regional Committee for Medical and Health Related Research Ethics in Western Norway), but exempted from review. Research on anonymous data not collected by the researchers themselves are exempted.
